# Biomarkers in Contrast-Induced Acute Kidney Injury: Towards A New Perspective

**DOI:** 10.3390/ijms25063438

**Published:** 2024-03-19

**Authors:** María Ángeles González-Nicolás, Cristian González-Guerrero, Marian Goicoechea, Lisardo Boscá, Lara Valiño-Rivas, Alberto Lázaro

**Affiliations:** 1 Renal Physiopathology Laboratory, Department of Nephrology, Instituto de Investigación Sanitaria Gregorio Marañón, Hospital General Universitario Gregorio Marañón, 28009 Madrid, Spain; rengac@yahoo.es (M.Á.G.-N.); cristian.gonzalez.guerrero@gmail.com (C.G.-G.); 2 Department of Nephrology, Hospital General Universitario Gregorio Marañón, 28009 Madrid, Spain; marian.goicoechea@gmail.com; 3 Instituto de Investigaciones Biomédicas Alberto Sols-Morreale (CSIC-UAM), 28029 Madrid, Spain; lbosca@iib.uam.es; 4 Centro de Investigación Biomédica en Red en Enfermedades Cardiovasculares (CIBERCV), Melchor Fernández Almagro 6, 28029 Madrid, Spain; 5 Department of Physiology, School of Medicine, Universidad Complutense de Madrid, 28040 Madrid, Spain

**Keywords:** contrast-induced acute kidney injury, biomarkers, contrast media, nephrotoxicity

## Abstract

Contrast-Induced Acute Kidney Injury (CI-AKI) remains a frequent iatrogenic condition since radiological procedures using intra-vascular iodinated contrast media (CM) are being widely administered for diagnostic and therapeutic purposes. Despite the improvement of the medical healthcare system worldwide, CI-AKI is still associated with direct short-term and indirect long-term outcomes including increased morbidity and mortality, especially in patients with underlying pre-existing renal function impairment, cardiovascular disease, or diabetes that could rapidly progress into Chronic Kidney Disease. Although the RIFLE (Risk, Injury, Failure, Loss, End-Stage Kidney Disease), AKIN (Acute Kidney Injury Network), and KDIGO (Kidney Disease Improving Global Outcomes) clinical criteria and recommendation guidelines are based on traditional “gold standard” biomarkers known as serum creatinine, glomerular filtration rate, and urinary output, new reliable serum and urinary biomarkers are still needed for an effective unified diagnostic strategy for AKI. Starting from previous and recent publications on the benefits and limitations of validated biomarkers responding to kidney injury, glomerular filtration, and inflammation among others, this review unravels the role of new emerging biomarkers used alone or in combination as reliable tools for early diagnosis and prognosis of CI-AKI, taking into account patients and procedures-risk factors towards a new clinical perspective.

## 1. Introduction

Acute Kidney Injury (AKI) is a silent and under-recognized syndrome whose morbidity and mortality worldwide is little recognized today. During AKI, as already mentioned, occurs a rapid and acute loss of renal function and it is also a risk factor for chronic kidney disease (CKD) development and progression to end-stage renal disease (ESRD) [1]. Nowadays, there is no effective pharmacological treatment for AKI and current approaches allow us to slow down the process, but not to prevent damage or avoid progression to ESRD. An estimated 13.3 million AKI episodes occur every year worldwide, 85% of which occur in the least developed or developing countries, representing a serious financial problem for the majority of people who need it, with less than 10% of patients in these settings receiving kidney replacement therapy. Many people cannot afford kidney treatment at all, resulting in the death of over 1 million people annually from untreated kidney failure [2,3].

Iodinated contrast drugs are widely used in imaging techniques for diagnostics and surgical procedures, enhancing the difference between body tissues in the images [4]. Advances in radiodiagnostics and angiography have led to improved clinical diagnosis and safe treatment of patients in recent years and most of these modern radiological techniques involve the administration of contrast media (CM) including those used for trauma and stroke diagnosis in the emergency department, cancer diagnosis, staging and follow-up, cardiovascular and peripheral vascular interventions, gastrointestinal, urological, and interventional pain procedures [5,6]. At present, millions of doses of intravascular CM are being administered worldwide. However, it is well established that CM exposure causes iatrogenic renal function impairment, which incidence is highly increasing, especially, in subjects with preexisting cardiovascular, diabetes, or renal disease. Therefore, CM-associated kidney dysfunction variates from slight serum creatinine (SCr) increase to severe AKI [7,8,9]. AKI secondary to CM injection has historically been called contrast-induced nephropathy (CIN) or contrast-induced AKI (CI-AKI). Worryingly, the enormous burden of CM used in contemporary clinical practice explains why CI-AKI is one of the top leading forms of hospital-acquired renal disease, being in fact, the third most common cause of AKI [10]. To date, CI-AKI is defined as a rise in SCr of 0.5 mg/dL (or higher) or 25% (or higher) from baseline, occurring within the 2–3 days after the intravascular injection of iodinated radiographic CM, that cannot be attributed to other direct sources [7,8,11,12,13,14,15,16]. Additionally, AKIN (Acute Kidney Injury Network) and KDIGO (Kidney Disease Improving Global Outcomes) guidelines suggested a rise in SCr ≥ 0.3 mg/dL with oliguria after hospitalization as a new standard to follow [12,17,18]. Since SCr is less accurate, it is better to define CI-AKI as a decrease in SCr clearance or estimated Glomerular Filtration Rate (eGFR) by 30–60 mL/min [7,13,14,15]. In some cases, CI-AKI may cause a more severe renal function impairment with progressive oliguria, requiring dialysis, which is associated with high mortality [7,13,16].

CI-AKI is responsible for approximately 10% of all cases of iatrogenic renal disease and although its overall incidence is low (estimated between 1% to 6%), however, this rate increases critically in high-risk populations, where mortality is also higher [7,8,9,12,13,14,15,19]. Consequently, it is necessary to early identify those high-risk patients to avoid post-procedure renal function impairment, to guide precise therapeutic management, and hence, to improve patient outcomes. Besides, there is an extremely narrow window for intervention, increasing the risk of fatality. Importantly, the use of CM in certain procedures, such as percutaneous coronary interventions (PCI) for acute coronary syndrome poses a risk of developing CI-AKI of 19% to 50% [20].

All of these data highlight that the identification and selection of specific urinary and/or serum biomarkers for the early diagnosis of renal cell damage rather than functional impairment due to their high sensitivity and specificity may overcome the limitations of SCr and urinary output (UO) as the “*gold standard*”, normalizing markers of CM-associated renal failure.

## 2. Pathophysiology of AKI and CI-AKI

AKI is characterized as multifactorial in origin and it is associated with complex pathophysiological mechanisms. In low- and middle-income countries, infections and hypovolemic shock are the predominant causes of AKI. In contrast, in more developed countries AKI occurs mainly in older hospitalized patients and is associated with sepsis, drug use (such as CM), or invasive procedures. AKI associated with infections and trauma is common in all regions of the world [21]. Depending on the origin of the damage, AKI could be classified as pre-renal, due to reduced renal perfusion, renal or parenchymal when renal structures (glomeruli, interstitium, tubules, or vessels) are directly affected, or postrenal or obstructive when there is an obstruction in urinary flow [22]. It is therefore difficult to establish a common physiological mechanism for all possible AKI, due to the different causes that can produce them, with complex mechanisms, and different combinations of effects. But the result is always common to all of them, a rapid decline in in kidney function [1,21,22]. In pre-renal AKI, the kidney tries to maintain extracellular volume and perfusion to vital organs, reabsorbing water and sodium, which results in oliguria. Consequently, the kidney does not eliminate solutes correctly and accumulates in the blood. In contrast, in post-renal AKI the increased pressure in the urinary tract is transmitted to the glomerulus, and when the pressure in the urinary space exceeds the filtration pressure, glomerular filtration is abolished.

On the other hand, in parenchymal AKI, and specifically in those caused by nephrotoxic drugs (such as CM) and/or nephrotoxins, sepsis or ischemia, inflammation, oxidative stress and apoptosis and cell death are the main pathological processes involved in renal failure [22,23]. In particular, each nephrotoxic drug is different and will act differently, although they will all affect and damage different cell structures. For example, cyclosporine acts directly on the mitochondria of proximal tubular cells by inhibiting the opening of the mitochondrial transition pore leading to an increase in intramitochondrial Ca^2+^, decreased mitochondrial volume, and ultimately cell death by apoptosis [23]. Tacrolimus has the same mode of action as cyclosporine, inhibiting inducible nitric oxide synthase (iNOS) and promoting renal vasoconstriction. Aminoglycosides such as gentamicin enter tubular cells via endocytosis, leading to phospholipidosis, which is closely related to cell death. Gentamicin activates proapoptotic proteins that directly damage mitochondria, thereby triggering apoptotic pathways, the production of reactive oxygen species (ROS), and an inflammatory response [24]. Cisplatin is a chemotherapeutic agent whose mechanism of nephrotoxicity has been extensively studied. It activates several pathways of apoptosis, affecting death receptors (Fas/FasL), mitochondria, and the reticulum. Cisplatin increases ROS levels that directly induce renal damage to cellular components including lipids, proteins, and DNA, and directly damage mitochondria, linking its effect to apoptosis. There is also an activation of TNF-α and NFΚB, leading to the amplification of damage by the production of proinflammatory cytokines and cellular infiltration, which aggravates renal damage [25,26]. Nephrotoxicity caused by paracetamol treatment is initially mitochondria-independent and is mainly focused on the endoplasmic reticulum (ER) of proximal tubular cells [27]. The presence of paracetamol triggers a stress response in the ER leading to increased expression and activation of the transcription factor GADD153 and activation of caspase 12, which will lead to activation of effector caspases 3 and 9 responsible for carrying out the apoptosis process [27].

**Figure 1:**
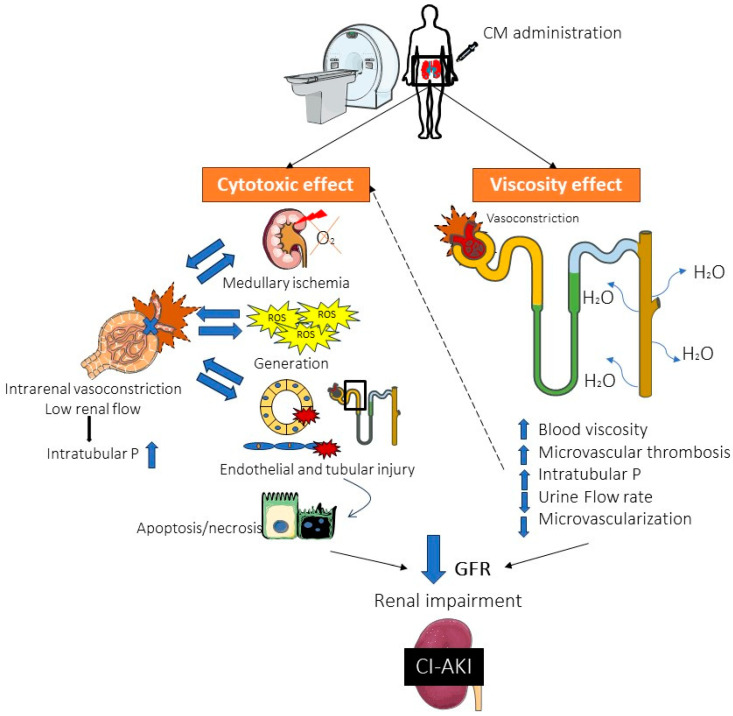
Contrast-induced acute kidney injury (CI-AKI) pathophysiology. Contrast media (CM) mediates, on the other hand, direct renal tubular and endothelial cytotoxicity, leading to increased intrarenal pressure and consequent increase of intratubular pressure, resulting in a vicious cycle of medullary hypoxia, oxidative stress and increased loss of function, integrity, and cell death. On the other hand, the viscous properties of CM trigger vasoconstriction increase blood viscosity, which reduces urinary flow rate, decreases microvascularization, and increases the risk of microvascular thrombosis. All this leads to an abrupt loss of renal function resulting in CI-AKI. P, pressure; ROS, reactive oxygen species; GFR, glomerular filtration rate.

Renal hemodynamic changes and nephrotoxic effects occur immediately after intravascular administration of iodinated contrast drugs and could last many hours to days. These unwanted side effects could be aggravated by intrinsic CM properties like osmolality and viscosity, but also due to concentration and volume administration. Generally, CM produces a rapid non-physiological vasodilatation followed by a long vasoconstriction, resulting in a prompt decrease in renal flow. This process results in a vicious cycle of medullary ischemia which causes, in turn, ROS generation and consequently, vascular endothelial and tubular injury [7,13]. Direct effects of kidney injury from exposure to CM are due to tubular epithelium toxicity substantiated by disruption of cell integrity, leading to loss of function, apoptosis, and eventually, necrosis. CM also increases blood viscosity, decreases microcirculation, and decreases urine flow rate, which increases the time that CM remains in the body and could produce microvascular thrombosis. All of this leads to an abrupt decrease in GFR, therefore, in kidney function [7,14,16] (Figure 1). Notwithstanding the above, the diagnosis of CI-AKI in clinical practice could be catastrophically delayed, as it is radically based on the identification of a late elevation of SCr and/or a decrease in eGFR. Consequently, this delay in diagnosis which places patients at high risk of progressive kidney damage, highlights the critical need to identify other blood and/or urine-sensitive and specific biomarkers of incipient tubular injury, leading to rapid prognosis and diagnosis of CI-AKI development.

## 3. Traditional “*Gold Standard*” Markers

Traditionally, the RIFLE (Risk, Injury, Failure, Loss, End-Stage Kidney Disease), AKIN, and KDIGO guidelines recommend the use of classic markers for AKI diagnosis and management. However, increased levels of these markers suggest functional changes, not kidney damage [28]. Despite this, the reliability of these markers is questioned and still controversial due to their lack of specificity and sensitivity, and high variability [29]. Worryingly, when these parameters are measurable in serum or urine, at least, 50% of the renal function may be lost [30]. Taken together, these reasons compel us to consider these obsolete markers as “data normalizers” for new accurate biomarkers of kidney injury.

### 3.1. Serum Creatinine (SCr)

Although in 80% of cases of CI-AKI the SCr starts to rise in the first 24 h after CM exposure, the SCr usually peaks 2–5 days after CM and returns to baseline or near baseline within 1–3 weeks. However, the diagnosis of CI-AKI is commonly based on an absolute (≥0.5 mg/dL) or relative (≥25%) increase in SCr within 48–72 h after intravascular CM administration [11]. These data imply that patients who develop CI-AKI can be treated late or, on the contrary, the prolonged hospital stay of patients who will never develop CI-AKI. Even so, changes in SCr are used to estimate acute changes in renal function (but does not allow us to see the existence of kidney damage) and SCr monitoring, remains the cornerstone for CIN diagnosis. Unfortunately, this confirms that SCr is the most widely used and accepted biomarker by nephrologists and the scientific community. However, SCr is not an adequate marker of renal function, mainly because there is no correlation between SCr and actual kidney function under nonstationary conditions [28,31]. In addition, Scr, as a CI-AKI biomarker, also has other limitations. Creatinine muscle production varies with age, sex, race, and weight, which affect muscle mass. Thus, daily changes in serum creatinine poorly reflect changes in kidney function in patients with AKI. On the other hand, urine creatinine not only comes from glomerular filtration, as renal tubules also secrete it, which means that changes in sCr will underestimate the actual fall in glomerular filtration rate (GFR). Some drugs could also alter creatinine tubular secretion. Besides this, the lower the GFR, the less creatinine is excreted. Consequently, the non-excreted creatinine is distributed in total body water, increasing serum levels. Thus, although CM decreases GFR almost immediately, is not until 24–48 h later that this is reflected in a Scr increase, making it not an effective biomarker [28]. For all these reasons, SCr becomes a retrospective, late, insensitive, and misleading measure of kidney damage [28,30,32].

### 3.2. Glomerular Filtration Rate (GFR)

Commonly, renal function is defined as the filtration capacity of the kidney, which could be expressed as GFR. However, GFR does not cover all kidney function which also involves glomerular permeability, tubular function, and several specific functions, such as vitamin D metabolism and erythropoietin production [33]. In addition, GFR could be estimated (eGFR) based on the empirical Cockcroft-Gault, MDRD (Modification of Diet of Renal Disease) or CKD-EPI (Chronic Kidney Disease Epidemiology Collaboration) formulas [34,35], which involve SCr levels as a common variable. Currently, patients with CI-AKI are diagnosed with an eGFR of 30–60 mL/min [13]. However, the latter issue is of particular importance, as changes in SCr lag behind changes in GFR, leading to the possibility of progression of AKI stage despite actual improvement in renal function, a shortcoming that could be overcome with the availability of real-time assessment of GFR [36]. For this reason, the aforementioned concerns about SCr may also apply to the determination of GFR, as the interpretation of changes using one parameter alone is difficult.

Vijayasimha et al. carried out a study to evaluate whether kidney injury molecule-1 (KIM-1) allows the identification of CI-AKI earlier than the rise in Scr. The study involved 100 consecutive patients with normal Scr undergoing angiographic procedures. Urinary KIM-1 was assessed at 4 h, 8 h, and 24 h after the angiographic procedure, while SCr was measured at basal, 24 h, and 48 h after the procedure. In CI-AKI a significant increase in urinary KIM-1 was observed after 24 h of coronary angiography (using iodixanol or iopromide), while there were no changes in estimated GFR up to 48 h after the angiography, indicating that GFR is not a good predictor for early stages of CI-AKI [29]. However, two recent clinical trials established the primary endpoint of drug efficacy by iohexol plasma clearance-based GFR and by serum cystatin-based (eGFRecys), respectively [37,38]. 

### 3.3. Urinary Output (UO)

Nephrologists recognized UO as the most sensitive system for determining CI-AKI. A “good” urine output (4150 mL/h) in the 6 h after the radiological procedure has been associated with reduced rates of AKI [39] and surprisingly, in a retrospective study, UO captured >40% AKI results than SCr [40]. Due to some limitations, it is not regularly used in the clinical setting, as it requires regular urine collection and catheterization of the patient.

## 4. Novel Biomarkers: The Necessity for a Precise CI-AKI Diagnosis

**Figure 2:**
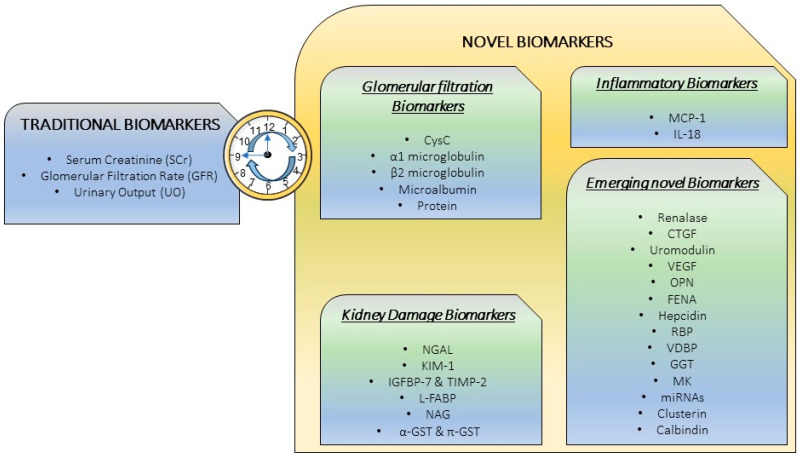
Traditional and novel biomarkers classification. *CysC*, Cystatin C; *NGAL*, Neutrophil Gelatinase-Associated Lipocalin; *KIM-1*, Kidney Injury Molecule-1; *IGFBP*, Insulin-like Growth Factor-Binding Protein; *TIMP*, Tissue Inhibitor of Metalloproteinase; *L-FABP*, Liver Fatty Acid-Binding Protein; *NAG*, N-Acetyl-β-D-Glucosaminidase; *GST*, α-Glutathione S-Transferase; *MCP-1*, Monocyte Chemoattractant Protein-1; *IL*, Interleukin; *CTGF*, Connective Tissue Growth Factor; *VEGF*, Vascular Endothelial Growth Factor; *OPN*, Osteopontin; *FENA*, Fractional Excretion of Sodium; *RBP*, Retinol Binding Protein; *VDBP*, Vitamin D Binding Protein; *GGT*, Gamma-glutamyltransferase activity; *MK*, Midkine; *miRNAs*, microRNA.

In recent decades, the identification of new biomarkers of AKI has been the subject of interest by scientists worldwide. However, the predictive, diagnostic, and prognostic ability of biomarkers in the context of iodinated contrast administration has been less studied. In recent years, many additional potential biomarkers have been newly described for the early detection of tubular dysfunction/lesion associated with CM administration, to reliably measure CI-AKI, and thus prevent patient outcomes. Some of them are well characterized and categorized and could be divided into different groups in response to different physiological conditions: some involved in glomerular filtration, others related to the inflammatory response and tubular cell injury or with a not well-defined relationship with the disease and new emergent biomarkers under study. Figure 2 schematically represents the traditional and novel biomarkers described for CI-AKI.

### 4.1. Glomerular Filtration Biomarkers

#### 4.1.1. Cystatin C (CysC)

CysC is a small molecule freely filtered by the glomerulus, and subsequently reabsorbed and metabolized by the proximal tubule [41]. Compared with SCr, serum CysC concentration (sCysC) is less dependent on age, gender, muscle mass, and nutrition and, therefore, more reliably predicts deterioration of renal function, becoming a sensitive marker of kidney injury. An increase in sCysC levels reflects a decrease in GFR and, therefore, there is an inherent delay between renal injury and a detectable increase in its levels [42]. Thus, sCysC increases more rapidly than SCr when GFR decreases [28,29]. CysC is usually undetectable in the urine of patients with normal renal function; however, tubular injury could result in measurable urinary levels, suggesting urinary CysC as a potential biomarker for AKI [42,43]. Regarding CI-AKI, CysC presented an excellent diagnostic specificity and negative predictive value in diagnosis of CI-AKI. It might be used as a tool for excluding CI-AKI in clinical practice. 48 h CysC performed better than 24 h CysC in CI-AKI diagnosis, among which a 15% increment achieved relatively the best diagnostic value [44].

The study of CKD patients undergoing coronary (CA) or peripheral angiography demonstrated that increased sCyC is a reliable marker to rule out CI-AKI and an independent predictor of death and dialysis [28,45,46]. Complementary studies in patients undergoing coronary angiography and/or therapeutic PCI showed that sCysC is incremented 24 h after CM application, and this correlated with CI-AKI and was associated with a higher incidence of adverse events at one year [14,47,48,49,50,51]. Complementarily, other studies state that sCyC can detect AKI 1–2 days earlier than Scr, and its sensitivity was also significantly higher than that of Scr. It was found that the sensitivity of CysC for diagnosing renal insufficiency was 82.8%, while that of Scr was only 68.2%, thus CysC is easier to detect. Therefore, as a marker of kidney function, CysC is more ideal than Scr [52,53]. Finally, a recent publication stated that sCysC at 24 h was the best biomarker for CIN diagnosis, while baseline levels of other common biomarkers were the best predictors of prognosis [54].

#### 4.1.2. α1-Microglobulin (α1-m)

α1-m is a member of the lipocalin family of proteins that is synthesized in the liver, freely filtered by glomeruli, and reabsorbed by the renal proximal tubular cells, where it is catabolized. The α1-m form is stable and, under normal conditions, appears very poorly filtered in the final excreted urine [55]. Therefore, urine levels above the reference values could indicate proximal tubular damage, resulting in α1-m being a preferred biomarker.

In one study of hospitalized patients with AKI secondary to coronavirus disease-2019 (20% in Europe of patients with COVID-19 develop AKI) was observed that proteinuria and α1-m excretion were higher in hospitalized patients who subsequently develop AKI compared with those who did not, and as mentioned above, prolonged hospital stay is a risk factor of developing CI-AKI due to the high likelihood of these patients undergoing diagnostic tests using CM. The median α1-m excretion was higher among AKI patients who experienced progression of their AKI stage compared with those who did not. In contrast, median proteinuria levels did not differ between AKI progressors and AKI non-progressors. These data show that in hospitalized patients who have developed secondary AKI, α1-m appears to be a good biomarker for the prediction of AKI, as its levels increase significantly with AKI. Furthermore, higher excretion is associated with a worse prognosis and increased mortality [56].

On the other hand, Afili WM et al. measured serum α1-m in 33 patients with no history of kidney disease before 6 h and 24 h after CM administration. This study revealed that serum measured α1-m was higher in patients with AKI following CM compared to patients without AKI, with a sensitivity and specificity of serum α1-m at 6 h after CM administration of 91.1% and 93.9%, respectively. However, at 6 h there were no statistically significant differences in creatinine levels. These data indicated that serum α1-m can be used as an early biomarker of contrast-induced nephropathy instead of serum creatinine as it rises 24 h, before any change in the serum creatinine [57,58]. Furthermore, a recent study concluded that α1-m is a suitable biomarker for early-stage kidney injury detection after cardiac surgery [59].

#### 4.1.3. β2-Microglobulin (β2-m)

β2-m is a small protein expressed on the cell surface of every nucleated cell and which is filtrated by the glomerulus; almost all β2-m proteins have undergone reabsorption and catabolism by the proximal tubular cells [29]. Increased levels of β2-m excretion in urine have been described as an early sign of tubular dysfunction due to many causes, such as exposure to nephrotoxic substances, cardiac surgery, and renal transplantation, preceding by 4–5 days the rise in serum creatinine levels.

In Zhu H et al. study, β2-m was upregulated in CI-AKI patients who underwent coronary angiography [60], and additionally, it was seen that in patients undergoing Coronary Computed Tomography Angiography (CCTA), serum β2-m showed values superior to creatinine-based parameters and similar with CysC, demonstrating that useful biomarker for the prediction and prognosis of CI-AKI at pre and early post-CCTA [61]. These observations are supported by recent publications that suggest that β2-m could be a suitable biomarker for predicting prognosis in CI-AKI [54,58,62]. However, the disadvantage of β2-m as a biomarker is its instability in the urine and fast degradation in room temperature and urine with pH < 6.0 [63].

#### 4.1.4. Microalbuminuria

The term microalbuminuria indicates urinary albumin at a concentration that is below the threshold for albumin detection by conventional measurement protocols and its value ranges between 30–300 mg/L [29]. It is suggested to be an important marker of glomerular structure and function alterations and it was designated as a biomarker to investigate the attenuation of CI-AKI by N-acetylcysteine [64]. Other authors, studying the impact of microalbuminuria complicated with low estimate glomerular filtration rate (eGFR) on the incidence and prognosis of contrast-induced CI-AKI in patients with coronary artery disease after coronary intervention have suggested that microalbuminuria complicated with low eGFR levels may increase the risk of CI-AKI and adverse cardiovascular events in patients after coronary intervention. Microalbuminuria could be a preventive biomarker to be taken into account for a preventive intervention. Microalbuminuria could be a preventive biomarker to be taken into account for a preventive intervention [65].

#### 4.1.5. Proteinuria

Increased urinary protein excretion (proteinuria) is the result of alterations in the glomerular filtration barrier, generally associated with specific podocyte damage. Therefore, total urinary protein has been highlighted as a diagnostic marker and as a predictive factor for progressive loss of renal function in clinical and non-clinical settings [66,67,68]. Specifically, in CI-AKI it has been demonstrated that proteinuria is an important risk factor and an independent indicator of 1-year mortality in patients with cerebrovascular disease [69]. However, as a biomarker for CI-AKI has a number of limitations, particularly in terms of prognosis. This has been demonstrated in a study about hospitalized patients with COVID-19 who develop secondary AKI, proteinuria levels did not differ between AKI-CKD progressors and AKI-CKD non-progressors, and it was also not a good predictor of mortality since no significant changes in its levels were seen in patients who died compared to those who did not, unlike other biomarkers [56].

### 4.2. Inflammatory Biomarkers

#### 4.2.1. Monocyte Chemoattractant Protein 1 (MCP-1)

MCP-1 is a potent chemotactic factor for monocytes and macrophages involved in ischemic and toxic AKI. However, its potential use as a biomarker has been less well studied. Several studies have shown that urinary MCP-1 expression (uMCP-1) is increased in both murine models and in patients with kidney injury [70]. Additionally, recent studies have shown that uMCP-1 in combination with other markers (e.g., epidermal growth factor –EGF-, KIM-1, neutrophil gelatinase-associated lipocalin –NGAL-) appears to represent a potent diagnostic and prognostic biomarker of tubulointerstitial injury and repair [30,70,71,72]. Therefore, MCP-1 alone or in combination with other molecules could be considered a useful biomarker in AKI, which could also be extrapolated to CI-AKI. In fact, in an experimental CI-AKI model, 5 days after CM injection MCP-1 protein levels were found to be elevated in the renal tubules of CI-AKI rats, accompanied by increased concentrations of IL-6 and TNF-α in the kidneys and the serum, ROS production, cell death, renal dysfunction and an increased excretion of other urinary AKI biomarkers. Although MCP-1 appears to be a promising biomarker for CI-AKI, data are currently only available in experimental models, not in human trials, and only once CI-AKI has already occurred. Therefore, despite existing data on the correlation between MCP-1 levels and the diagnosis and prognosis of tubulointerstitial damage and repair, little is known about its efficacy in the diagnosis and prognosis of AKI and/or in humans [73].

#### 4.2.2. Interleukin 18 (IL-18)

IL-18 is a pro-inflammatory cytokine released by proximal tubular epithelial cells in response to injury. After kidney injury, IL-18 is massively secreted into the urine and increases in the first 6 to 12 h before a significant decline in renal function and remains elevated for up to 48 h [42]. Human studies demonstrated that urinary IL-18 (uIL-18) levels were significantly higher in patients with AKI than in those without AKI at 24 and 48 h before SCr increased [68,74,75]. Surprisingly, a similar study in elderly patients undergoing gadolinium-enhanced magnetic resonance imaging (MRI) displayed an increase in uIL-18 after CM administration for 24 h, compared to a delayed increase in SCr levels [76]. However, several clinical performed to evaluate new diagnostic biomarkers of CI-AKI following CA or PCI, indicated that no statistically significant differences in uIL-18 were observed in patients with CI-AKI compared to patients without CI-AKI; neither were significant changes observed between uIL-18 before and after the intervention [77,78]. Similarly, Zdziechowska M et al. have shown that patients who underwent planned or emergency coronary angiography and received contrast agent serum IL-18 levels were analyzed at baseline, after 24 and 72 h from angiography. No significant differences were observed between marker levels in patients who developed CI-AKI and those who did not. After 24 h, serum levels of IL-18 were higher in patients with CI-AKI, however, this difference was on the verge of significance. In addition, after 72 h IL-18 decrease in serum can be seen, acting as an ineffective biomarker [79].

### 4.3. Kidney Damage Biomarkers

#### 4.3.1. Neutrophil Gelatinase-Associated Lipocalin (NGAL)

NGAL is a protein of the lipocalin family that covalently binds to neutrophil gelatinase and is dramatically upregulated in the kidneys after ischemic or toxic damage [80,81]. NGAL has been shown to decrease apoptosis and increase tubular cell proliferation, favoring renal tissue recovery after damage. This particular role may explain the sustained elevated urinary NGAL (uNGAL) levels observed during the days following renal injury [82]. Therefore, NGAL may also be detected in the proximal tubular epithelium due to the failure of reabsorption of filtered NGAL [83].

Surprisingly, NGAL increases rapidly in a dose-dependent manner and is detectable at early points (3 h after kidney damage and peaks at 6–12 h). Moreover, its elevation could persist markedly for up to 5 days if the initial injury is severe. For this reason, among all new biomarkers, NGAL is one of the most widely investigated, also in the setting of CI-AKI [32].

One of the most ambitious studies based on large cohorts of patients, both children, and adults, receiving contrast agents and undergoing cardiac catheterization, intra-arterial coronary angiography, or computed tomography (CT), revealed a very good performance of plasma and uNGAL in the prediction of CI-AKI [84,85]. More recently, uNGAL monitoring studies revealed that NGAL also predicted the severity of CI-AKI into CKD and/or hemodialysis progression, demonstrating that NGAL is potentially superior compared to conventional markers of nephropathy after invasive coronary procedures with contrast agents [86,87,88,89,90].

With respect to its measurement in serum (sNGAL), the result is similar. In the previous article of Zdziechowska et al., it was observed that after 24 h serum levels of biomarkers KIM-1 and IL-18 were insignificantly higher in a group with CI-AKI. Significant changes in levels in time (baseline vs. 24 h vs. 72 h) were observed only for NGAL [79]. In another study, serum NGAL was measured in patients with CI-AKI before PCI in patients with coronary artery disease. Compared with patients without CI-AKI, baseline serum NGAL was higher in patients with CI-AKI, although Scr and estimated eGFR were not different between groups. Patients in the highest tertile of baseline serum NGAL showed a significantly higher rate of major adverse cardiac and cerebrovascular events. These data indicate that baseline serum NGAL is a reliable marker for predicting CI-AKI, and high serum NGAL levels are associated with a higher incidence rate of long-term adverse events after [91].

However, the ANTI-CI-AKI clinical trial study concluded that uNGAL was not able to predict CI-AKI and was an inadequate tool to guide an early intervention strategy to prevent CI-AKI outcomes [92] and perhaps further studies will be necessary.

#### 4.3.2. Kidney Injury Molecule 1 (KIM-1)

KIM-1 is a type I transmembrane glycoprotein that is poorly expressed in the kidneys of healthy subjects. However, following ischemia/reperfusion injury (IRI) and nephrotoxic exposure, KIM-1 is dramatically overexpressed in the proximal tubular epithelium in both rodent models [93,94] and humans [95]. Following kidney injury, the extracellular domain of KIM-1 is released from the tubular epithelial cells in a metalloproteinase-dependent manner. This release, in combination with the persistent increase of renal KIM-1 synthesis, leads to a rapid increase in exclusively urinary KIM-1 after 48 h [96].

Recent clinical studies have shown that urinary KIM-1 concentration (uKIM-1) is higher in patients with AKI due to ischemia and hypoperfusion, nephrotoxins, CIN nephropathy, and cardiac surgery compared to controls, and also that it is a predictor of the risk of developing AKI [97,98,99]. Since KIM-1 is defined as an effective early biomarker in AKI, several rodent models showed that uKIM-1 concentration was elevated 12 h following CM administration [100,101]. Furthermore, differences in uKIM-1 production were found in pre-existing kidney injury models of hypertension, diabetes, and nephropathy subjected to CM administration [102]. Multiple human clinical studies confirm the potential use of uKIM-1 as an early valuable marker for CIN diagnosis. In a large study of more than 3000 patients undergoing CA, KIM-1 levels were found to be significantly increased in the urine of CIN patients at the sixth hour compared to baseline [103]. These results confirmed a previous study in which KIM-1 was measured 12 h after cardiac catheterization in humans and exhibited a good predictive value for CI-AKI diagnosis with high sensitivity and specificity [104]. For all these reasons, KIM-1 seems to have a high predictive value for the diagnosis of CI-AKI.

#### 4.3.3. Insulin-Like Growth Factor-Binding Protein 7 (IGFBP-7) and Tissue Inhibitor of Metalloproteinases-2 (TIMP-2)

IGFBP-7 and TIMP-2 are master regulatory biomarkers of G1 cell cycle arrest, predominantly expressed in proximal and distal tubular cells in response to DNA damage. Unlike many previously studied biomarkers, both are markers of cellular stress in an early phase of tubular cell damage caused by a wide variety of triggers and could act as a protective mechanism for cell injury [105,106]. Remarkably, these two proteins are discussed in combination because some preclinical studies demonstrated their superiority when used together to predict advanced AKI stages compared to other biomarkers [107].

In 2014, the FDA approved the first point-of-care device (NephroCheck test) that allows the determination of both urinary IGFBP-7 and TIMP-2 in critically ill patients with an increased risk of developing moderate-to-severe AKI. The NephroCheck test anticipates the diagnosis of AKI, therefore reducing the social and health care costs associated with worsening disease in these patients [108,109]. Surprisingly, other clinical and observational studies reported that the combined IGFBP-7/TIMP-2 test was the strongest predictor of AKI and significantly improved the risk assessment after major surgery [109]. Despite this, the impact of CM on these biomarkers and the evaluation of the potential development of CI-AKI have been little studied. A prospective clinical trial study performed on 172 children aged 0–18 years with the intravascular injection of CM was investigated in order to determine whether they can diagnose CI-AKI early. Changes in urinary NGAL, IGFBP-7, and TIMP-2 were measured. CI-AKI occurred in 14.59%. In the CI-AKI group, urinary levels of NGAL, IGFBP-7, TIMP-2 and [IGFBP-7]*[TIMP-2] were significantly increased 2 h after angiography and remained at high levels at 6 h. This increase was faster than SCr. When both urinary IGFBP-7 and TIMP-2 were used together the specificity was better than either marker alone and this combination has a good clinical value for the early diagnosis of CI-AKI in children and it could be extrapolated to the adult population [110].

#### 4.3.4. Liver Fatty Acid-Binding Protein (L-FABP)

L-FABP is a protein that is expressed in several tissues, but predominantly localizes to the renal proximal tubule epithelium and is excreted into the tubular lumen before binding to peroxisomal toxic products [111,112,113]. Urinary L-FABP (uL-FABP) has been extensively studied in preclinical and clinical models and has been suggested as a potential biomarker in a variety of pathological conditions, such as AKI, CKD, diabetic nephropathy, IgA nephropathy, and CI-AKI [114,115].

Nakamura et al. demonstrated that uL-FABP increase was associated with CIN. Remarkably, before angiography, uL-FABP concentration was significantly higher in 13 of 66 patients suffering from CIN compared to the non-CIN group or healthy volunteers [116], suggesting that uL-FABP levels could clinically serve as a useful predictive biomarker to detect CI-AKI onset before CM exposure. In another clinical trial to assess CI-AKI in patients after percutaneous cardiovascular procedures it was observed that L-FABP introduction into patient care resulted in a statistically significant improvement of 4.6%. Results indicated that the inclusion of L-FABP was 2.9 times more likely to correctly identify patients’ risk for AKI and were more than twice as likely to treat for AKI by providing volume expansion and withholding nephrotoxic medications. The study showed the greatest clinical utility in the pre-procedure and peri-procedure settings but limited value in the post-procedure setting [117].

#### 4.3.5. N-acetyl-β-d-glucosaminidase (NAG)

NAG is a lysosomal enzyme that is expressed mainly in the brush border of proximal renal tubular cells but also is localized in the liver, brain, and spleen [118]. Due to its nature, NAG does not cross the glomerular barrier and, therefore, urinary NAG release reflects tubular impairment.

Increased NAG levels have been described in multiple AKI onsets showing, in all cases a high sensitivity for detecting AKI [118,119,120,121]. However, increased NAG levels have been reported in a variety of non-clinical AKI conditions, limiting the use of NAG as a marker of AKI in some settings [122].

Comparative investigations suggested that NAG is also a valuable biomarker in CI-AKI, exclusively in patients undergoing CA and/or therapeutic PCI. A robust study showed that urinary NAG (uNAG) and sCr were significantly increased on days 1 and 2 after radiocontrast injection, compared to patients without CI-AKI. Moreover, uNAG levels peaked earlier and increased more than sCr levels [123]. Clinical studies in children corroborated that uNAG detection may contribute to a more accurate diagnosis of CI-AKI by indicating increased stress on the kidneys after CA [124,125]. For all this, NAG could act as a promising marker of CI-AKI.

#### 4.3.6. α,π glutathione S-Transferase (α-GST, π-GST)

α-GST and π-GST are promising proximal and distal tubular cytosolic enzymes respectively, that are considered promising biomarkers for early detection of AKI. In AKI, a single urinary π-GST measurement performed better than α-GST at predicting dialysis requirement or death, whereas α-GST performed better in patients with AKI in stage 1 or 2. However, neither marker had good prognostic discrimination [126]. Finally, further other investigations showed that urinary GSTs predicted AKI progression or hospital mortality after cardiovascular surgery, where CM administration is correlated with kidney damage following an increase in urinary biomarkers [127,128,129].

### 4.4. Emerging Novel Biomarkers

Among the classical CI-AKI biomarkers, a large number of promising molecules have been constantly investigated to assess their predictive and intrinsic diagnostic value in a wide spectrum of renal disorders, including CI-AKI, to improve patient outcomes. However, some of them are exclusively studied in animal models although some are not yet in CI-AKI models. Hence, there is an open need to discover, evaluate, and validate the best-performing biomarkers in clinical trials, and, consequently, to extrapolate them to clinical practice. These include:

#### 4.4.1. Renalase

Renalase is a novel catecholamine-metabolizing amine oxidase synthesized mainly by proximal tubular cells and secreted into the urine and bloodstream, which plays a key role in blood pressure regulation [130]. In animal models, renalase-deficient mice displayed a severe IRI reflected by higher SCr and a significant increase in tubular inflammation, apoptosis, and necrosis, which was partially alleviated by renalase pretreatment [131]. Renalase was also documented to ameliorate cisplatin-induced AKI [132].

Singularly, a recent study of ioversol-induced CI-AKI showed an exceptional protective effect of renalase pretreatment on renal function and kidney morphology in rats [133]. To date, only one clinical study is available to evaluate the urinary renalase concentration in patients with preserved renal function undergoing CA/PCI interventions, in which it was observed that their urinary levels were reduced after these interventions [133]. However, further studies will be required to validate renalase as a diagnostic marker of CI-AKI and also to assess that the exogenous recombinant renalase administration could serve as a preventive therapeutic agent [134,135].

#### 4.4.2. Connective Tissue Growth Factor (CTGF)

CTGF is an important profibrotic factor that is upregulated in kidney diseases. When endogenous CTGF is artificially suppressed in experimental models of kidney injury, renal function is restored [136]. In a murine model of CI-AKI, CTGF gene expression levels increased 2 days after CM administration in nephrectomized mice compared to the non-CM infusion group [137]. Despite this, clinical correlations have not yet been addressed for CI-AKI so we cannot predict its diagnostic and prognostic ability for CI-AKI and further studies will be necessary to evaluate CTGF protein as a kidney injury biomarker in humans in this regard.

#### 4.4.3. Uromodulin

Uromodulin, also known as Tamm-Horsfall protein, is a glycoprotein localized mainly in renal tubular cells lining the thick ascending limb of the loop of Henle. In the last decade, uromodulin has been implicated in the pathophysiology of several diseases, including AKI and CKD, showing a protective role through the downregulation of interstitial inflammation [138]. Surprisingly, several publications expressed an exponential interest in the role of uromodulin in CM nephrotoxicity, showing a huge urinary decrease after CM administration in patients, but also in canine experimental models [139,140,141]. Recent publications have demonstrated that a lower uromodulin-to-creatinine ratio is associated with AKI after cardiac surgery. Overall, the consideration of uromodulin as a biomarker in clinical measures could be considered to minimize the risk of developing AKI/CI-AKI [142,143].

#### 4.4.4. Vascular Endothelial Growth Factor (VEGF)

VEGF is an endothelial-specific growth factor that promotes endothelium proliferation, differentiation, and survival and mediates vasodilatation, microvascular permeability, and matrix remodeling. In the kidney, it is prominently expressed in tubular epithelial cells and glomerular podocytes and is associated with the pathophysiology of diabetic nephropathy, glomerulosclerosis, and tubule-interstitial fibrosis [144].

Despite the lack of updated clinical results regarding CI-AKI, a single study of CM administration in rats suggested that VEGF protein levels in kidney tissue were significantly higher compared to controls and consequently, it was correlated with advanced kidney damage. Interestingly, paricalcitol pretreatment ameliorated kidney injury markers, including VEGF levels, thus offering the opportunity to prevent CI-AKI [145]. Wang Y et al. examined renal changes after CI-AKI development in a diabetic rabbit model, such as HIF-1α and VEGF expression at different time points after the administration of the CA. SCr and blood urea nitrogen (BUN) concentrations reached their maximum values on day 3 in the diabetic rabbits with the contrast agent group, whereas they were slightly increased without statistical differences after the iohexol administration in the healthy rabbits with the contrast agent group [146]. All this indicates that VEGF could act as an early biomarker for the diagnosis and prognosis of CI-AKI. However, further studies in humans are needed.

#### 4.4.5. Osteopontin (OPN)

OPN is a glycoprotein expressed in bone, immune, smooth muscle, epithelial, and endothelial cells and plays an important role in bone mineralization and resorption. In addition, OPN is found in the kidneys and urine and its expression results elevated in several renal pathologies [147], suggesting its potential role as a novel biomarker. Besides this, clinical trials and observational studies demonstrated that OPN had a positive association with the risk of AKI in patients undergoing CA [148] and could predict the patient outcome after cardiac arrest [149]. In a clinical trial performed in individuals undergoing coronary and/or peripheral angiography 4 candidate biomarkers for AKI were included (KIM-1, IL-18, OPN, and CysC). In adjusted models, neither KIM-1 nor IL-18 improved discrimination for CI-AKI; the addition of OPN and CysC to the CI-AKI clinical model significantly increased statistical significance (from 0.69 to 0.73, *p* for change < 0.001) and would facilitate risk stratification for future cardiorenal outcomes or CKD progression. These data suggest that OPN may act as a biomarker, in combination with other biomarkers, for the diagnosis and prognosis of CI-AKI [150].

#### 4.4.6. Fractional Excretion of Sodium (FENa)

FENa is the percentage of the sodium filtered by the kidney that is excreted in the urine. Principally, under normal conditions, most of the sodium is reabsorbed by intact tubular cells, but another small part is excreted in the urine. Therefore, FENa could be calculated to assess changes in renal blood flow and kidney tubular damage under pathological conditions [151].

In a clinical study, it was observed that patients with oliguric AKI presented <1% FENa in pre-renal AKI and >3% in acute tubular necrosis (ATN), demonstrating the applicability of FENa as a prognostic marker [151]. Subsequently, different studies were conducted to evaluate the effects of CM exposure in human kidneys, indicating that CM administration is associated with increased urinary FENa [152,153]. These findings were also corroborated in animal models [154,155].

#### 4.4.7. Hepcidin

Hepcidin is a small peptide hormone principally produced in hepatocytes, but also in other tissues such as the kidney or brain. In the kidney, hepcidin is freely filtered by the glomeruli with significant tubular uptake and catabolism, which could be excreted in the urine and also found in plasma [156]. Surprisingly, clinical researchers determined that lower levels of hepcidin could predict the risk for AKI development [157,158,159]. However, in patients undergoing PCI, serum hepcidin concentration increased at 4 and 8 h after CM administration, while urine hepcidin was significantly lower in PCI patients with CI-AKI compared with baseline [160]. Consequently, early changes in hepcidin could predict a later CI-AKI onset contributing to early patient risk stratification.

#### 4.4.8. Retinal Binding Protein (RBP)

RBP is a carrier protein filtered mainly by the glomeruli and reabsorbed by the proximal tubules of the kidney. The relation of variation in serum RBP levels was initially studied in a small cohort of patients with kidney disease and healthy volunteers, in whom the urinary concentration of RBP was differentially excreted, leading to the conclusion that RBP could be useful for the diagnosis and monitoring of kidney disease progression [161]. Accordingly, in a randomized controlled study, urinary RBP concentration was rapidly elevated in patients undergoing peripheral arterial angiography [162]. Likewise, in a miniature pig model of CI-AKI (one single dose of iohexol after dehydration) BUN, Scr, serum, and urinary RBP and β2-m levels were measured. The urinary RBP level of the Iohexol group peaked on day 5 but remained higher than that of the Sham group by day 8. The serum RBP and β2-m levels of the iohexol group peaked on day 3 and then slowly declined but remained higher than those of the Sham group by day 8. These results in combination with human studies showed a promising role of RBP in the diagnosis and prognosis of CI-AKI [163].

#### 4.4.9. Vitamin D Binding Protein (VDBP)

VDBP is filtered in the glomerulus, reabsorbed by proximal tubule epithelial cells, and subsequently catabolized, thus reducing urinary excretion. After tubular injury, increased urinary VDBP (uVDBP) levels are expected to be found, reflecting renal damage. Chaykovska et al. demonstrated that uVDBP is a potent biomarker of major renal adverse effects after CA intervention, concluding that uVDBP concentration 24 h after CM administration was predictive for dialysis and death. Correction of uVDBP concentrations for creatinine excretion confirmed its predictive value and was consistent with increased levels of urinary KIM-1 and baseline plasma creatinine in patients with the above-mentioned complications, being a promising biomarker of CI-AKI and associated events even until 90 days after CM exposure [164].

#### 4.4.10. Gamma Glutamyl Transferase (GGT)

GGT is an enzyme localized in the brush border of the renal proximal tubules that appears in the urine (uGGT) when damage occurs. Early clinical studies focused on tubular toxicity due to CM exposure showed a relevant early increase in uGGT enzyme activity in patients undergoing radiological examination with intravascular injection of CM [165]. Recently, the same results were obtained in patients undergoing PCI, where urinary GGT showed a predictive value for CI-AKI progression [166]. However, more research is required to demonstrate its effectiveness.

#### 4.4.11. Midkine (MK)

MK is a heparin-binding growth factor that regulates inflammation, cell growth, survival, and migration. In the kidney, MK is expressed in both proximal and distal tubular epithelial cells. Strikingly, MK was found to be significantly elevated 2, 4, and 8 h after CM exposure, exclusively in patients undergoing PCI, and then, returned to baseline after 24 h and started to decrease after 2 days [167]. These data are in agreement with the study by Ahmed M et al. [168], suggesting MK determination as an early predictor of CI-AKI in patients with Acute Coronary Syndrome and as a great opportunity for CI-AKI treatment.

#### 4.4.12. MicroRNAs (miRNAs)

miRNAs are short, non-protein-coding RNA molecules that regulate gene expression at the post-transcriptional level by mRNA repression and are involved in multiple cell mechanisms, such as proliferation, differentiation, death, or inflammation. For this reason, miRNA differential expression is principally related to pathophysiological conditions, including kidney injury [29,169], and they have been proposed for early diagnosis in CI-AKI [170]. Among others, miR-21 has been extensively studied in the kidney, where it plays a crucial role in cell proliferation and downregulation of apoptosis after renal IRI [171], while serum and urine levels of miR-21 also predict AKI in cardiac surgery patients [172]. In particular, different preclinical studies that were validated in humans revealed that circulating levels of miR-188, miR-30a, miR-30c, and miR-30e should be considered as early biomarkers in CI-AKI [173].

#### 4.4.13. Clusterin

Clusterin is a sulfated glycoprotein mainly present in the cytoplasm of proximal and distal convoluted tubules [174]. In the context of kidney injury (nephrotoxicity, ischemia, surgery) in experimental models, it has been suggested that clusterin could play an anti-apoptotic role in favoring cell protection and regeneration [175].

In a variety of nephrotoxic models including cisplatin, higher urinary levels of clusterin outperformed BUN and SCr for detecting proximal tubular injury even in the absence of functional effects and was correlated with positive clusterin kidney immunostaining [175,176]. Interestingly, in ischemia-reperfusion models, clusterin deficiency worsens renal inflammation and kidney fibrosis after IRI, suggesting a crucial role in kidney cell repair and proliferation [177,178]. In a rat model in which CM was injected at different points after left nephrectomy, clusterin was identified by proteomics as a differentially increased protein after CI-AKI among other known CI-AKI biomarkers such as KIM-1, β2-m or IGFBP-7 [179]. Despite the lack of studies indicating the predictive potential value of clusterin in CI-AKI, a controlled clinical trial performed by Da et al. has been recently published, suggesting for the first time that clusterin could detect early nephrotoxicity and predict AKI when compared AKI cases with non-AKI controls [180]. These studies open the possibility of using clusterin as a future biomarker in CI-AKI.

#### 4.4.14. Calbindin

Calbindin is an intracellular protein involved in the regulation of calcium reabsorption that is commonly expressed and localized in the distal tubule and the proximal part of the collecting ducts. As a kidney injury marker, calbindin has been studied in rat and sheep nephrotoxic models, thus reflecting changes in urinary excretion without histological alterations [181,182]. In humans, calbindin concentration was significantly higher in urine from patients receiving cisplatin chemotherapy while SCr levels remained unchanged [183,184]. Unfortunately, no data are currently available on its expression in CI-AKI, and future studies will have to be developed to demonstrate its effectiveness as a biomarker in this regard.

Additional information on these traditional and novel biomarkers, providing data on their specific location, where and with which techniques they are currently measured, as well as the advantages and disadvantages of their current use; the great potential for their future use in the early determination of CI-AKI is summarized in Table 1.

## 5. Combination of Biomarkers: A Future Approach

To date, most preclinical and clinical studies on kidney diseases only validate one biomarker rather than a combination of several, limiting the ability to determine whether several specific panels of biomarkers are more specific and predictive than one biomarker alone. Biomarker combinations could be integrated into clinical practice using viable high-throughput technology to increase the ability to detect and diagnose multiple renal disorders early, unravel new pathophysiological mechanisms, and, consequently, prevent undesirable long-term patient outcomes.

Vaidya et al. suggested that comparative values of multiple urinary biomarkers detection (NGAL, Hepatocyte Growth Factor (HGF), VEGF, Protein, and KIM-1) were associated with sensitive and specific prognosis and diagnosis of AKI in humans [122]. Furthermore, in a cross-sectional study, the combination of urinary Matrix Metalloproteinase 9 (MMP-9), KIM-1, and NAG markers allowed AKI diagnosis earlier than an increase in SCr [97]. However, the results of the SAPHIRE study demonstrated that performing exclusively urinary TIMP-2/IGFBP7 significantly improved AKI risk stratification, thus providing additional information on clinical variables [107]. In a cohort study based on major surgeries, it was proposed that urinary NGAL and L-FABP determination may improve the diagnostic yield of AKI [185]. In addition, the combination of urinary IL-18 and KIM-1 concentrations had a very good predictive value for predicting AKI stage 3 or death, thus identifying high-risk patients after cardiac surgery [186]. Moreover, in studies on obstructive nephropathy and cisplatin-induced nephrotoxicity, the combined detection of the EGF/MCP-1 ratio, urinary NGAL, and urinary KIM-1 outperformed that of any of the biomarkers alone in predicting progressive renal damage, thus, avoiding undesirable long-term outcomes [71,72]. Finally, several recent investigations focusing on contrast-induced nephrotoxicity deciphered multiple biomarker combinations that successfully predicted early CI-AKI, predominantly in patients undergoing CA. While Wybraniez et al. proposed that post-procedural determination of urinary KIM-1 and IL-18 predicted CI-AKI; Connolly et al. suggested that serum L-FABP and plasma NGAL were the best combination to predict early CI-AKI [78,187]. Finally, Banda et al. stated that serum CysC at 24 h was the best biomarker for CIN diagnosis, while baseline levels of serum IL-18, β-2M, and TNFα were the best for predicting prognosis [54].

## 6. Conclusions

Iodinated contrast agents are widely used in various clinical procedures, increasing the risk of patients developing mild to severe renal changes and CI-AKI. In the absence of current pharmacological treatments for AKI, intensive care is the only possible supportive tool and new novel diagnostic approaches are urgently needed.

However, there is no consensus among experts on which panel of biomarkers is the most appropriate one for the diagnosis and prognosis of AKI or CI-AKI. Serum creatinine is not the ideal biomarker for AKI for several reasons. Firstly, creatinine production does not always reflect a steady state and, secondly, it is the product of creatine and phosphocreatine metabolism in muscle cells, so its levels depend on age, gender, and diet. In addition, creatinine is slow to accumulate and is a delayed marker rather than a specific marker of renal or tubular injury. Many studies have suggested the potential use of biomarkers to predict CI-AKI, such as NGAL, IL-18, or L-FABP, but the data are currently inconclusive as to the most sensitive and specific biomarker.

Biomarker assessment of CI-AKI, combined and integrated with relevant clinical information, should allow tailoring the management and treatment of AKI to individual patients at an early stage, with the potential to improve clinical practice, save costs, and reduce the risk of progression to CKD.

## Figures and Tables

**Table 1:** Characteristics of traditional and novel biomarkers of contrast-induced acute kidney injury.

Biomarker	Location	Sample	Advantages	Disadvantages	Cutoff for CI-AKI Prediction	Detection Methods
Creatinine	Glomerulus	Blood/urine	* Routine measurement * Cheap	* Depends on age, gender, muscle mass and nutrition * No correlation with renal function in acute situations * Decreases with intensive serum therapy	≥0.3 mg/dL at 48 h or 50% above baseline over the next 7 days, or by urine volume reduction of 0.5 mL/kg/h for 6 h	* Enzymatic assay * Automated biochemical analyzer
CysC	Glomerulus Proximal tubule	Blood/urine	* Early increase and related to renal function * The best diagnostic marker for CI-AKI	Not routinely available in all laboratories	≥10% increase at 24 h	* ELISA * Nephelometric and turbidimetric assays
α_1_-m	Glomerulus Proximal tubule	Urine	* Considered as a sensitive indicator of impaired renal function * Associated with increased risk of kidney disease progression and all-cause mortality	Not routinely available in all laboratories	NA	* Immuno-nephelometric assay * Automated biochemical analyzer
β_2_-m	Glomerulus Proximal tubule	Blood/urine	* Routine measurement * Difference if the damage is of glomerular or tubular origin	* It is not a diagnostic test for any specific disease* Should be measured in blood and urine along with other renal function tests	>1.26 mg/dL at baseline	* ELISA * Nephelometric assays
Proteinuria	Glomerulus	Blood/urine	* Routine measurement * Cheap	Not a good prognostic or mortality biomarker	NA	* Colorimetric methods * Automated biochemical analyzer
MCP-1	Renal cells	Urine	Reports the presence of inflammation	Not routinely used	NA	* ELISA * Bio-Plex
IL-18	Proximal tubule Distal tubule Distal collecting tubule Tubular cells	Urine	* Early increase in AKI before renal function deterioration * Strong predictor of AKI in the subsequent 48 h * Sensitive biomarker of AKI especially post-cardiopulmonary bypass	* Not routinely used * Its use in CI-AKi is inconclusive	≥25% increase at 24 h	* ELISA * Bio-Plex
NGAL	Glomerulus Proximal tubule Distal tubule	Blood/urine	* Increases its urinary concentration before creatinine * Predicts diagnosis and prognosis of CI-AKI	Not routinely used	≥20 ng/mL uNGAL; ≥179 sNGAL	* ELISA * Immunoblotting * Turbidimetric assays
KIM-1	Proximal tubule	Blood/urine	* Very sensitive and specific marker of proximal tubular kidney injury * Distinguishes ischemic acute tubular necrosis from prerenal azotemia	Not routinely used	>0.425 ng/mL	* ELISA * Immunoblotting
IGFBP7xTIMP-2	Renal epithelial cells	Urine	* Strong predictor of AKI * Its early detection could facilitate new therapeutic and protective strategies	* Not validated in CI-AK * Not routinely available in all laboratories	> 0.3 is a strong predictor of AKI and a value > 2.0 indicates higher kidney stress and probable AKI within 12–24 h	NephroCheck^®^
L-FABP	Proximal tubule	Urine	* Useful predictive biomarker to detect CI-AKI onset before CM exposure * Sensitivity for predicting the need for dialysis	Not routinely used	≥24.5 µg/g	ELISA
NAG	Proximal tubule Lysosomal enzymes	Urine	* Correlates with histological evidence of renal proximal tubule damage * Sensitive marker of tubular injury in AKI and CI-AKI	* Increased levels have been reported in a variety of non-clinical AKI conditions * Limited use as an AKI marker	NA	* ELISA * Spectrophotometric assays
α-GST/π-GST	Proximal tubule Distal tubule	Urine	Promising biomarkers for early detection of AKI	* Not routinely used * Limited studies in CI-AKI	NA	* ELISA * Bio-Plex
Renalase	Proximal tubule	Blood/urine	In animal models, its increase is related to renal improvement	* Not routinely used * Limited studies in CI-AKI	NA	ELISA
CTGF	All tissues	Urine	Important profibrotic biomarker upregulated in AKI	* Not routinely used * Limited studies in CI-AKI	NA	ELISA
Uromodulin	Distal tubule Loop of Henle	Urine	Lower Uro/Crea ratio is associated with AKI after cardiac surgery	* Not routinely used * Limited studies in CI-AKI	NA	* ELISA * Bio-Plex
VEGF	Glomerulus Proximal tubule	Blood	Its increase is related to advanced kidney damage	* Not routinely used * Limited studies in CI-AKI	NA	* ELISA * Bio-Plex
OPN	Proximal tubule Loop of Henle Distal tubule	Blood	It is associated with the risk of AKI in patients undergoing CA	Not routinely used	NA	* ELISA * Bio-Plex
Hepcidin	Glomerulus	Blood/urine	Early changes could predict a later CI-AKI onset	Not routinely used	NA	* ELISA * Dot blot * SELDI-TOF MS
RBP	Glomerulus Proximal tubule	Blood/urine	* Rapidly elevated, useful for the diagnosis and monitoring of kidney disease progression * Biomarker of proximal tubular dysfunction. Used as a diagnostic tool in some proximal tubulopathies and renal interstitial diseases	Not routinely available in all laboratories	NA	ELISA
VDBP	Glomerulus Proximal tubule	Urine	Possible predictor of dialysis use and CM-associated death.	Not routinely used	NA	ELISA
GGT	Proximal tubule	Urine	Predictive value for CI-AKI progression	Not routinely used	NA	ELISA
Midkine	Proximal tubule Distal tubule	Blood	Early increase in CI-AKI	Not routinely used	NA	ELISA
miRNAs	Preinjury biomarkers	Blood/urine	Early potential biomarkers for CI-AKI	* Not routinely used * The role of circRNAs or lncRNAs in CI-AKI remains unknown * More studies are needed to verify the putative miRNA-mRNA pairs in CI-AKI, as well as the interaction mechanisms and downstream signaling pathways	NA	* RT-qPCR * RNA-seq technology
Clusterin	Proximal tubule Distal tubule	Urine	* Increases after treatment with CM * Early detection of nephrotoxicity and prediction of AKI	* Not routinely used * Few studies in CI-AKI	NA	Bio-Plex
Calbindin	Distal tubule Collecting duct tubule	Urine	Elevated levels of nephrotoxicity	* Not routinely used * No studies in CI-AKI	NA	* ELISA * Arrays * Bio-Plex

*CI-AKI*, Contrast-Induced Acute Kidney Injury; *CM*, Contrast Media; *CA*, Coronary Angiography; *CysC*, Cystatin C; *NGAL*, Neutrophil Gelatinase-Associated Lipocalin; *KIM-1*, Kidney Injury Molecule 1; *IGFBP*, Insulin-like Growth Factor-Binding Protein; *TIMP*, Tissue Inhibitor of Metalloproteinase; *L-FABP*, Liver Fatty Acid-Binding Protein; *NAG*, N-Acetyl-β-D-Glucosaminidase; *GST*, α-Glutathione S-Transferase; *MCP-1*, Monocyte Chemoattractant Protein 1; *IL*, Interleukin; *CTGF*, Connective Tissue Growth Factor; *VEGF*, Vascular Endothelial Growth Factor; *OPN*, Osteopontin; *RBP*, Retinol Binding Protein; *VDBP*, Vitamin D Binding Protein; *GGT*, Gamma-glutamyltransferase activity; *miRNAs*, MicroRNA; ELISA, Enzyme-Linked Immunosorbent Assay; *CircRNAs*, Circular RNAs; *lncRNAs*, Long non-coding RNAs; *NA*: There are no precise or clear data in the literature or there is controversy over the data.

## Data Availability

No new data have been created in this review article, only a review of the state of the art of biomarkers in acute renal failure-induced by iodinated contrast agents, and all the data noted have been extracted from the relevant literature in the article.
